# Reducing the CP content in broiler feeds: impact on animal performance, meat quality and nitrogen utilization

**DOI:** 10.1017/S1751731117000660

**Published:** 2017-05-02

**Authors:** P. Belloir, B. Méda, W. Lambert, E. Corrent, H. Juin, M. Lessire, S. Tesseraud

**Affiliations:** 1 URA, INRA, 37380 Nouzilly, France; 2 Ajinomoto Eurolysine S.A.S., F-75817 Paris Cedex 17, France; 3 EASM, INRA, 17700 Saint-Pierre-d’Amilly, France

**Keywords:** broiler, low-protein diet, performance, meat quality, environment

## Abstract

Reducing the dietary CP content is an efficient way to limit nitrogen excretion in broilers but, as reported in the literature, it often reduces performance, probably because of an inadequate provision in amino acids (AA). The aim of this study was to investigate the effect of decreasing the CP content in the diet on animal performance, meat quality and nitrogen utilization in growing-finishing broilers using an optimized dietary AA profile based on the ideal protein concept. Two experiments (1 and 2) were performed using 1-day-old PM3 Ross male broilers (1520 and 912 for experiments 1 and 2, respectively) using the minimum AA:Lys ratios proposed by Mack *et al*. with modifications for Thr and Arg. The digestible Thr (dThr): dLys ratio was increased from 63% to 68% and the dArg:dLys ratio was decreased from 112% to 108%. In experiment 1, the reduction of dietary CP from 19% to 15% (five treatments) did not alter feed intake or BW, but the feed conversion ratio was increased for the 16% and 15% CP diets (+2.4% and +3.6%, respectively), while in experiment 2 (three treatments: 19%, 17.5% and 16% CP) there was no effect of dietary CP on performance. In both experiments, dietary CP content did not affect breast meat yield. However, abdominal fat content (expressed as a percentage of BW) was increased by the decrease in CP content (up to +0.5 and +0.2 percentage point, in experiments 1 and 2, respectively). In experiment 2, meat quality traits responded to dietary CP content with a higher ultimate pH and lower lightness and drip loss values for the low CP diets. Nitrogen retention efficiency increased when reducing CP content in both experiments (+3.5 points/CP percentage point). The main consequence of this higher efficiency was a decrease in nitrogen excretion (−2.5 g N/kg BW gain) and volatilization (expressed as a percentage of excretion: −5 points/CP percentage point). In conclusion, this study demonstrates that with an adapted AA profile, it is possible to reduce dietary CP content to at least 17% in growing-finishing male broilers, without altering animal performance and meat quality. Such a feeding strategy could therefore help improving the sustainability of broiler production as it is an efficient way to reduce environmental burden associated with nitrogen excretion.

## Implications

Broiler production is known to produce large amounts of ammonia, contributing to eutrophication and soil acidification. One efficient way to limit ammonia emission from manure is to decrease nitrogen excretion by broilers by lowering the dietary CP content. We investigated the consequences of such a decrease associated with a supplementation of amino acids (AA) on broiler performance, meat quality and nitrogen utilization during the growing-finishing phase. Using the ideal protein concept, reducing dietary CP content from 19% to 17% decreased nitrogen excretion and volatilization without negative consequences on animal performance or meat quality.

## Introduction

The environmental effects of livestock production are well described in the literature (Steinfeld *et al*., 2006; de Vries and de Boer, 2010; Gerber *et al*., 2013). The main environmental burden caused by broiler production is ammonia emission, responsible for water pollution (eutrophication) and soil acidification (Bouwman *et al*., 2002; Méda *et al*., 2011). Ammonia is emitted from the manure by the breakdown of undigested protein and uric acid. As reported by Méda *et al*. (2011), a possible way to decrease ammonia emission is to decrease nitrogen excretion by lowering the dietary CP content. Indeed, several studies have shown a reduction in nitrogen excretion by about 10% with a 1 percentage point decrease in the dietary CP content in broilers (Aletor *et al.*, 2000; Bregendahl *et al*., 2002; Gomide *et al*., 2011).

Formulation of low CP diets requires controlling the provision of indispensable AA, that is, Lys, Met (+ Cys), Thr, Val, Ile, Leu, Trp, Arg, Phe (+Tyr) and His. In particular, the need for the first limiting indispensable AA can be satisfied by incorporating feed-grade AA. To balance the AA profile of the diet, practical formulation is generally based on an ‘ideal AA profile’ or ‘ideal protein concept’, for instance to maximize growth performance. The indispensable AA requirements in ideal protein are usually expressed relative to the requirement of Lys (National Research Council, 1994; Mack *et al*., 1999; Wu, 2014). However, contrary to what is classically observed in pigs (Gloaguen *et al*., 2014), broiler performance is not maintained systematically when the CP content in the diet is reduced, even when the indispensable AA requirements are apparently met (Aletor *et al.*, 2000; Bregendahl *et al*., 2002; Dean *et al*., 2006). These findings raise the question of the adequacy of AA provision, and also whether a requirement for indispensable AA or non-indispensable AA such as glycine and its precursors should be met (Dean *et al*., 2006, Siegert *et al*., 2015 and 2016). Moreover, few studies have been performed during the growing-finishing period (from 21 days of age) whereas this stage of production corresponds to >75% of total feed intake and is the most important in terms of economic (feed cost) and environmental impact (e.g. nitrogen excretion), and the consequences of a reduction in dietary CP content on meat quality are rarely documented. Therefore, the aim of the experiments presented here was to evaluate the effects of reducing the dietary CP content in growing-finishing broilers on animal performance, carcass composition, meat quality and nitrogen utilization (i.e. body retention, excretion and volatilization).

## Material and methods

Two successive dose response studies were performed. Experimental procedures and animal care were carried out according to current French legislation and under Authorizations 004601 and 006865 granted to S. T. and H. J., respectively, by the French Ministry of Agriculture, Agrifood and Forestry.

### Animals, experimental design and diets

A total of 3000 (experiment 1) and 2000 (experiment 2) 1-day-old Ross PM3 male chicks were reared together on sawdust in an experimental poultry unit from days 1 to 20 (INRA EASM, Le Magneraud, France for experiment 1 and INRA PEAT, Nouzilly, France for experiment 2). Broilers were fed the same starter and grower diets (Supplementary Table S1). They were wing-tagged individually at day 7. At day 21, before the experimental period, they were weighed. Among broilers with similar BW (i.e. within 1 SD), 1520 and 912 were randomly distributed in 40 and 24 pens of 3 m² (experiments 1 and 2, respectively), provided with sawdust (38 broilers/pen; 8 pens/treatment; 12 kg sawdust/pen). The average BW of the selected broilers was the same in both experiments (945±90 g). The ambient temperature programme applied was 31°C from days 0 to 3, 29°C from days 4 to 6, 28°C from days 7 to 13, 26°C from days 14 to 20, 24°C from days 21 to 24, 22°C from days 25 to 27 and 20°C from day 28 until the end of the experiment. The lighting schedule was of 23 h of light from days 0 to 3 and of 18 h of light from days 4 to 31. During the whole experiment, broilers had free access to water and feed. From days 21 to 35 (i.e. the experimental period), broilers were offered one of the experimental pelleted diets shown in Table 1. In both experiments, the experimental diets (Table 1) provided 13.2 MJ ME/kg and were sub-limiting in digestible Lys (dLys) at 0.9% (to be certain that the variations in measured responses are only due to the changes in dietary CP content). In each diet, the actual digestible AA (dAA) to dLys ratios were equal or above the ratios proposed by Mack *et al*. (1999), except for Arg and Thr. The minimum dArg:dLys ratio was 108% (instead of 112%) and the minimum dThr:dLys ratio was 68% (instead of 63%) based on recent literature data (Rostagno *et al*., 2011; Wu, 2014) and on results from a previous experiment showing that feed conversion ratio (FCR) could possibly be altered when decreasing the CP content (Supplementary Tables S2 and S3). Indeed, the dThr:dLys ratio proposed by Mack *et al*. (1999) was low in comparison with the recent recommendations. This is particularly important in low CP diets because the concentration of the dispensable AA Gly is reduced and, without Gly supplementation, additional Thr as a precursor of Gly may be required (Dean *et al*., 2006; Siegert *et al*., 2015 and 2016). The actual ratios of dArg:dLys and dThr:dLys in the experimental diets were therefore equal or above these new ratios (Table 1).

**Table 1:** Feedstuffs and chemical composition (%) of the diets differing in CP contents fed to broilers between 21 and 35 days of age

	Experiment 1					Experiment 2		
Diets	CP19%	CP18%	CP17%	CP16%	CP15%	CP19%	CP17.5%	CP16%
Feedstuffs								
Maize	43.4	47.2	51.1	54.9	58.7	43.4	48.4	54.6
Soyabean meal	28.2	24.6	21.1	17.5	13.9	28.2	23.7	17.8
Wheat	20.0	20.0	20.0	20.0	20.0	20.0	20.0	20.0
Soyabean oil	4.9	4.2	3.6	2.9	2.2	4.9	4.1	3.0
Dicalcium phosphate	1.7	1.7	1.7	1.7	1.7	1.7	1.7	1.7
Calcium carbonate	0.80	0.80	0.80	0.80	0.80	0.77	0.79	0.83
Elancoban 200	0.05	0.05	0.05	0.05	0.05	0.05	0.05	0.05
Mineral–Vitamin premix^1^	0.80	0.80	0.80	0.80	0.80	0.80	0.80	0.80
dl-Methionine	0.17	0.19	0.22	0.25	0.28	0.17	0.20	0.25
l-Lysine-HCl	0.05	0.16	0.27	0.37	0.48	0.05	0.18	0.36
l-Threonine	0.01	0.06	0.11	0.15	0.20	0.01	0.07	0.15
l-Arginine	–	0.08	0.17	0.25	0.33	–	0.005	0.20
l-Valine	–	0.04	0.09	0.13	0.18	–	0.02	0.11
l-Isoleucine	–	0.04	0.08	0.13	0.17	–	0.006	0.10
l-Tryptophan	–	0.01	0.02	0.03	0.04	–	–	0.02
Calculated composition								
AMEn (MJ/kg)	13.2	13.2	13.2	13.2	13.2	13.2	13.2	13.2
CP	19.0	18.0	17.0	16.0	15.0	19.0	17.5	16.0
Calcium	1.00	1.00	1.00	1.00	1.00	1.00	1.00	1.00
Available phosphorus^2^	0.40	0.40	0.40	0.40	0.40	0.40	0.40	0.40
Calculated digestible AA values^3^								
dLys	0.90	0.90	0.90	0.90	0.90	0.90	0.90	0.90
dMet	0.41	0.42	0.43	0.45	0.46	0.41	0.42	0.45
dMet+Cys	0.67	0.67	0.67	0.67	0.67	0.67	0.67	0.67
dThr	0.61	0.61	0.61	0.61	0.61	0.61	0.61	0.61
dVal	0.78	0.77	0.76	0.74	0.73	0.78	0.73	0.73
dIle	0.71	0.69	0.67	0.66	0.64	0.71	0.64	0.64
dLeu	1.42	1.34	1.26	1.18	1.10	1.42	1.32	1.19
dTrp	0.20	0.19	0.19	0.18	0.17	0.20	0.18	0.17
dArg	1.09	1.06	1.03	1.00	0.97	1.09	0.97	0.97
dPhe	0.85	0.79	0.72	0.66	0.59	0.85	0.77	0.66
dTyr	0.61	0.57	0.52	0.47	0.42	0.61	0.55	0.48
dPhe+Tyr	1.46	1.36	1.24	1.13	1.01	1.46	1.32	1.14
dHis	0.45	0.42	0.38	0.35	0.32	0.45	0.41	0.36
dSer	0.82	0.76	0.70	0.64	0.57	0.82	0.75	0.64
dGly	0.66	0.61	0.56	0.51	0.46	0.66	0.59	0.51
dSer+Gly	1.48	1.37	1.26	1.15	1.03	1.48	1.34	1.15
Calculated digestible AA ratio (% of dLys)^4^								
dMet	45	47	48	50	51	45	47	50
dMet+Cys	75	75	75	75	75	75	75	75
dThr	68	68	68	68	68	68	68	68
dVal	87	86	84	83	81	87	81	81
dIle	79	77	75	73	71	79	71	71
dLeu	158	149	140	131	122	158	147	132
dTrp	23	22	21	20	19	23	20	19
dArg	121	118	115	111	108	121	108	108
dPhe	95	88	80	73	66	95	86	74
dTyr	68	63	58	52	47	68	62	53
dPhe+Tyr	163	151	138	125	113	163	148	127
dHis	50	46	43	39	36	50	45	40
dSer	92	85	78	71	64	92	83	71
dGly	73	67	62	56	51	73	66	57
dSer+Gly	165	152	140	127	115	165	149	128

AA, amino acids.

1 Supplied per kilogram of diet: NaCl=3 g; Co=0.6 mg; Cu=20 mg; Fe=58 mg; I=2 mg; Mn=80 mg; Se=0.2 mg; Zn=90 mg; retinyl acetate=15 000 IU; cholecalciferol=4300 IU; dl-*α* tocopheryl acetate=100 mg; thiamine mononitrate=5 mg; riboflavin=8 mg; calcium pantothenate=25 mg; cyanocobalamin=0.02 mg; menadione=5 mg; pyridoxine hydrochloride=7 mg; folic acid=3 mg; biotin=0.3 mg; niacin=100 mg; choline chloride=550 mg; antioxidant (buthylhydroxyanisole, propyl gallat, ethoxyquin)=50 mg.

2 Available phosphorus was calculated from total phosphorus in feedstuffs and availability coefficients from Sauvant *et al*. (2004).

3 Digestible AA content was calculated from the total AA feedstuff content (chemical analyses) using digestibility coefficients from Sauvant *et al*. (2004).

4 In all diets, AA:Lys ratios were equal or above the ratios proposed by Mack *et al*. (1999): dMet+Cys:Lys=75%, dVal:dLys=81%, dIle:dLys =71% and dTrp:dLys=112%. For Thr and Arg, the minimum ratios were dThr:dLys=68% and dArg:dLys=108%.

For both experiments, the same feedstuff batches were used and analysed before formulation by Ajinomoto Eurolysine S.A.S for total AA contents (Supplementary Table S4). From these analyses, and using digestibility values of AA taken from Sauvant *et al*. (2004), dAA content of feedstuffs were calculated, and were used to formulate the experimental diets. In experiment 1, only the two extreme diets were formulated using linear programming to obtain 19% and 15% CP. The three intermediary diets (i.e. 18%, 17% and 16% CP) were obtained by blending these diets at different proportions (75:25, 50:50 and 25:75 for the 18%, 17% and 16% CP diets, respectively; Table 1). In experiment 2, the three experimental diets were formulated using linear programming to reach 19%, 17.5% and 16% CP, respectively (Table 1). Due to these differences in formulation strategy, the intermediary diets in experiment 1 contained the whole range of added feed-grade AA and a higher inclusion of these AA compared with experiment 2. As a result, some dAA concentrations were lower in the 17.5% and 16% CP diets of experiment 2 compared with the 17% and 16% CP diets of experiment 1. Moreover, Arg, Trp and Ile ratios to Lys in the intermediate diets of experiment 1 were above the levels recommended by the adjusted ideal AA profile of Mack *et al*. (1999).

### Animal performance, carcass characteristics and meat quality traits

Total feed intake (g) over the experimental period was measured per pen. At the end of the experiment, all broilers were weighed individually after 6 h of fasting. Average BW (g) and BW gain (g) were calculated for each pen. Feed conversion ratio was calculated for the experimental period from feed intake and BW gain. Four broilers per pen, representative of the average BW in the pen, were selected and slaughtered (32 broilers/treatment) in an experimental slaughter house. Broilers were electrically stunned in a water bath and then killed by ventral neck cutting. After partial evisceration (only the gut was removed), whole carcasses were air-chilled and stored at 2°C until the next day. Carcasses, abdominal fat and the left *Pectoralis* muscles (*P. major* and *P*. *minor*) were weighed and total breast meat weight was calculated ((*P. major*+*P*. *minor*)×2). Abdominal fat and breast meat yield were expressed as a percentage of BW.

In experiment 2, the ultimate pH of the *P. major* muscle was measured at 24 h *postmortem* with a portable pH meter (model 506; Crison Instruments SA, Alella, Barcelona, Spain) by inserting a glass electrode directly into the thickest part of the muscle. Breast colour was measured on the cranial ventral side of the muscle using a Miniscan spectrocolorimeter (HunterLab, Reston, VA, USA), and using the CIE LAB (international convention defined by CIE, Vienna, Austria) trichromatic system for lightness (L*) values. After being weighed at 24 h *postmortem*, the *P. major* muscle was placed in a hanging plastic bag and stored at 2°C for 96 h. After hanging, the sample was wiped with absorbent paper and weighed again. The difference in weight corresponded to the drip loss and was expressed as the percentage of the initial muscle weight.

### Nitrogen utilization

For each pen (*n*=8/treatment), total nitrogen intake (N_intake_, g) was calculated by multiplying total feed intake (FI) of the pen by CP content of the diet and divided by 6.25 (equation (1)). Whole-body nitrogen retention (N_ret_, g) was estimated according to equation (2) by multiplying the total BW gain of the pen by a constant value for whole-body nitrogen content (N_body_=29 g/kg; ITAVI, 2013) in agreement with previous studies (Aletor *et al*., 2000; Bregendahl *et al*., 2002) which showed that whole-body nitrogen content was not affected by a reduction in dietary CP content, even when dietary CP is limiting. The efficiency of nitrogen retention (N_effi_, %) was calculated using equation (3). Total excretion of nitrogen (N_exc_, g) was calculated according to the difference between N_intake_ and N_ret_ (equation (4)) and was also expressed per kg of BW gain using equation (5) (N_exc_BWG_, g/kg BW gain) to allow comparison between treatments and experiments:1
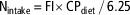

2
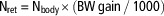

3
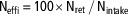

4
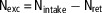

5
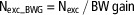



A sample of sawdust was collected before the experimental period, frozen and stored at −20°C. At day 35, four pens per treatment in each experiment were randomly selected. For those pens, total manure was removed and weighed (after broiler withdrawal), from which a sample of 1500 g was taken by mixing all the manure. During the experiments, no extra sawdust was added and no manure was removed. To stop all gaseous emissions from the manure, manure samples were frozen and stored at −20°C. Total nitrogen loss (N_loss_, g) through gas emissions was estimated for these pens using equation (6), W_manure_ being the total weight of the manure produced in the pen (kg), N_manure_ the nitrogen content of the manure (g/kg), W_sawdust_ the weight of sawdust used as bedding material (W_sawdust_=12 kg/pen) and N_sawdust_ the nitrogen content of the sawdust (g/kg). The nitrogen volatilization rate (N_vol_, %) was calculated using equation (7) and expressed as a percentage of total N excretion:6
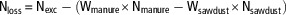

7
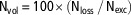



### Chemical analyses

Samples of the main feed ingredients and diets, sawdust and manure were analysed for dry matter (DM) (AFNOR method V18-109), nitrogen (Kjedahl method, ISO 1871) and AA content (only for the feed ingredients and diets, ISO 13903) by Ajinomoto Eurolysine S.A.S. Customers Laboratory (Amiens, France). The analysed values for nitrogen and AA content of the experimental diets (Supplementary Table S4) were consistent with expected values.

### Statistical Analyses

One-way ANOVA was performed using the GLM procedure of SAS (SAS Institute Inc., Cary, NC, USA) for BW, feed intake, BW gain, FCR, breast meat yield, abdominal fat (both experiments) and ultimate pH, lightness and drip loss (experiment 2). For BW, BW gain, feed intake and FCR the statistical unit was the pen (*n*=8/treatment). For breast meat yield, abdominal fat, ultimate pH, lightness and drip loss, the statistical unit was the animal (*n*=32/treatment). Normality of data was assessed using the Shapiro–Wilk test and the hypothesis for a normal distribution of the data was not rejected. Differences between treatments were tested and significance was accepted at *P*<0.05.

The relationships between N_effi_, N_exc_BW,_ N_vol_, nitrogen and moisture (i.e. 100 – DM) contents in the manure content *v*. CP content of the experimental diet were evaluated using linear regressions (R software v. 3.2.3; R Core Team, Vienna, Austria). In experiment 1, regression methods (i.e. linear, quadratic, linear plateau and curvilinear plateau models) were also used to model the effects of CP on animal performance and carcass characteristics using the REG procedures of SAS.

## Results

### Animal responses and consequences on carcass and meat quality traits

In experiment 1, reducing the dietary CP content from 19% to 15% did not alter BW, BW gain or feed intake, in contrast to FCR (*P*<0.01; Table 2). The FCR was significantly higher in the 16% and 15% CP diets compared with the other three diets. The breast meat yield was not affected by the dietary CP. The percentage of abdominal fat was increased by the reduction in dietary CP content (*P*<0.01), the highest values being observed for the 16% and 15% CP diets (Table 2). Only a linear relation between FCR and CP was found to be significant (FCR=1.95−0.017×CP; *R* ^2^=0.53; *P*<0.001).

**Table 2:** Performance and carcass characteristics of Ross PM3 male broilers fed with diets differing in CP between 21 and 35 days of age (experiment 1)

	Diets						
	CP19%	CP18%	CP17%	CP16%	CP15%	SEM	*P*-value
Performance^1^							
BW at day 35 (g)	2460	2470	2466	2451	2461	10.6	0.99
BW gain (g)	1479	1496	1494	1446	1478	12.1	0.72
Feed intake (g)	2430	2477	2472	2459	2528	16.9	0.48
Feed conversion ratio	1.64^b^	1.65^b^	1.65^b^	1.69^a^	1.71^a^	0.01	<0.01
Carcass characteristics^2^							
Breast meat yield (% of BW)	20.1	20.2	20.8	20.5	19.5	0.15	0.08
Abdominal fat (% of BW)	2.16^b^	2.30^ab^	2.45^a^	2.61^a^	2.51^a^	0.04	<0.01

1 *n*=8/treatment.

2 *n*=32/treatment. ^a,b^Values within a row with different superscripts differ significantly at *P*<0.05.

As shown in Table 3, no significant effect of dietary CP on animal performance (i.e. BW, BW gain, feed intake and FCR) was observed in experiment 2, even though a tendency was observed for feed intake (*P*=0.053). The percentage of abdominal fat was significantly increased (*P*=0.02) when decreasing CP content but with no effect on breast meat yield (*P*=0.06). The dietary CP decrease also had significant effects on meat quality traits with an increase in ultimate pH and a decrease of meat lightness and drip loss (*P*<0.001 for both ultimate pH and L*, and *P*=0.045 for drip loss).

**Table 3:** Performance, carcass characteristics and meat quality traits of Ross PM3 male broilers fed with diets differing in CP contents between 21 and 35 days of age (experiment 2)

	Diets				
	CP19%	CP17.5%	CP16%	SEM	*P*-value
Performance^1^					
BW at day 35 (g)	2288	2332	2324	12.4	0.32
BW gain (g)	1338	1380	1374	12.1	0.32
Feed intake (g)	2242	2323	2348	19.1	0.053
Feed conversion ratio	1.68	1.68	1.71	0.01	0.17
Carcass characteristics^2^					
Breast meat yield (% of BW)	20.8	20.9	21.4	0.10	0.06
Abdominal fat (% of BW)	1.93^b^	2.09^ab^	2.15^a^	0.04	0.02
Meat quality traits^2^					
Ultimate pH	5.92^c^	6.04^b^	6.14^a^	0.02	<0.001
Lightness	51.6^a^	49.5^b^	46.3^c^	0.30	<0.001
Drip loss (%)	3.07^a^	2.53^ab^	2.33^b^	0.13	0.045

1 *n*=8/treatment.

2 *n*=32/treatment. ^a,b,c^Values within a row with different superscripts differ significantly at *P*<0.05.

### Nitrogen utilization and manure composition

**Figure 1:**
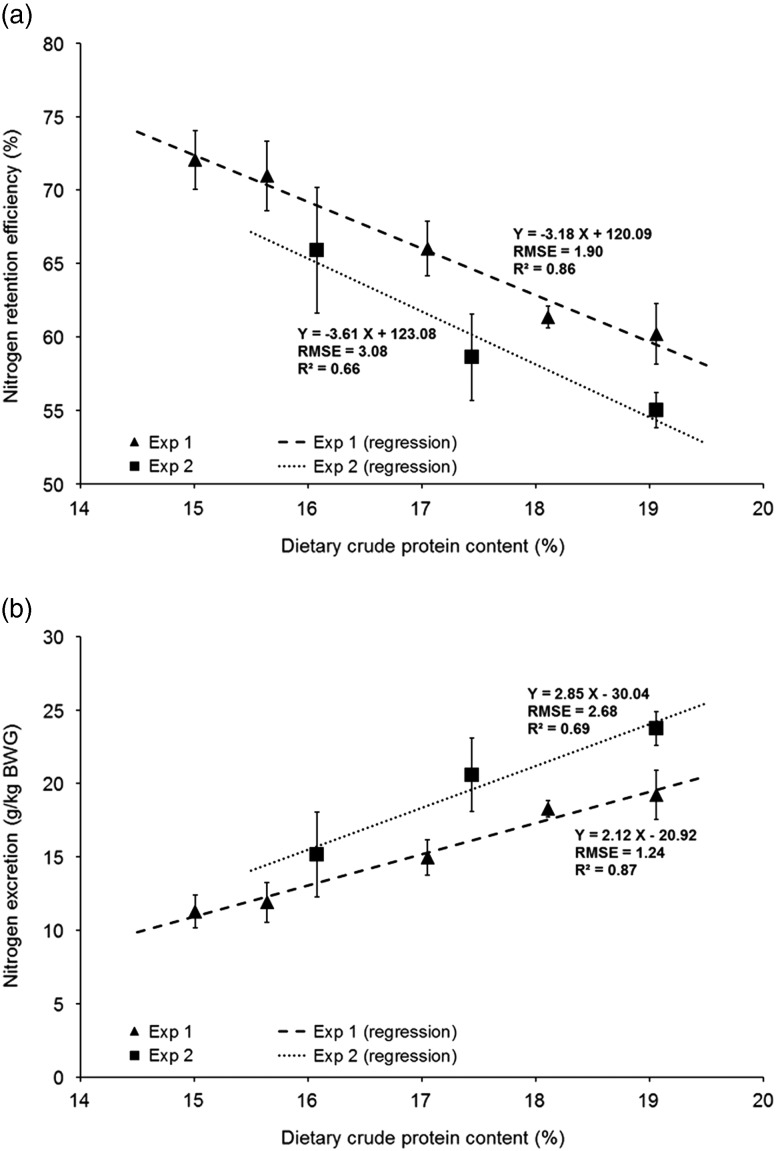
Nitrogen utilization in male Ross PM3 broilers according to the dietary CP content. Average values (±SD) of (a) the efficiency of nitrogen utilization (N_effi_, equation (3)) and (b) nitrogen excretion (N_exc_BWG_, equation (4)). Linear regressions were fitted with pen values (*n*=8/treatment).

**Figure 2:**
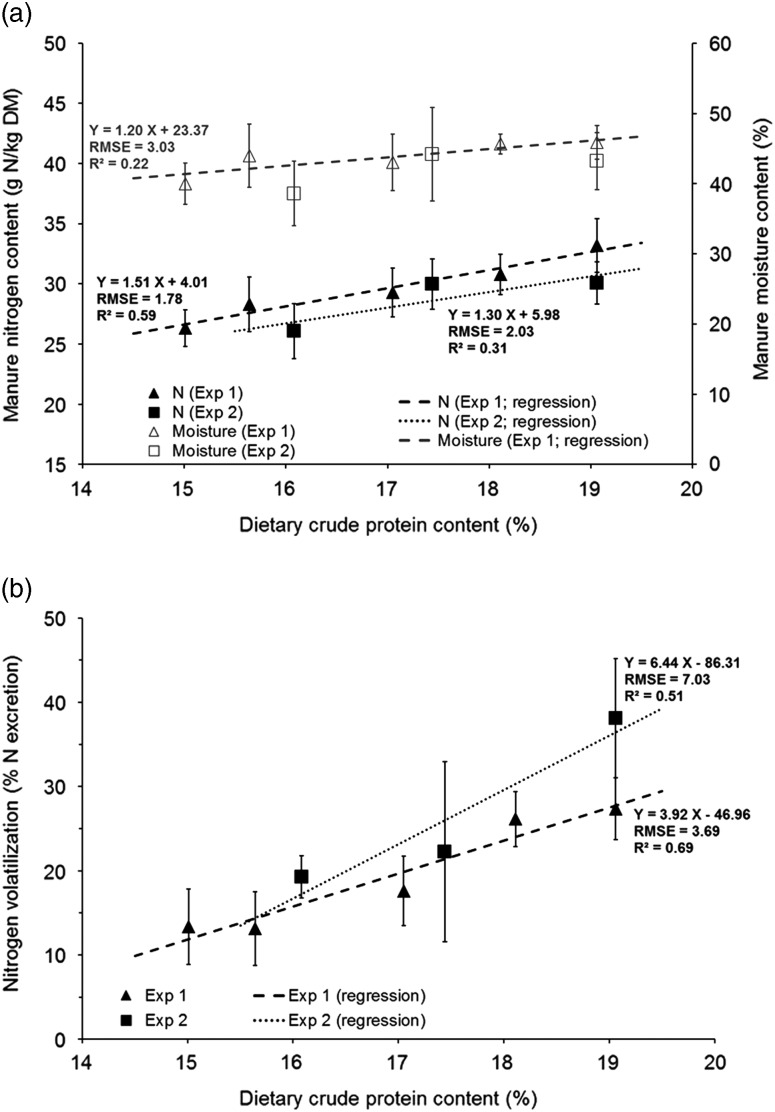
Manure composition and calculated nitrogen volatilization according to the dietary CP content. Average (±SD) values of (a) nitrogen and moisture content of manure and (b) total nitrogen volatilization (N_vol_, equation (7)). Linear regressions were fitted with pen values (*n*=4/treatment). For manure moisture in experiment 2, coefficients of the linear regression were not significantly different from 0.

The evolution of estimate efficiency of nitrogen retention, nitrogen excretion, nitrogen and moisture contents in the manure, and nitrogen volatilization with dietary CP content are given in Figure 1a, b, 2a and b, respectively. The efficiency of nitrogen retention increased from +3.2% to +3.6%/CP percentage point when dietary CP content was reduced (Figure 1a). Nitrogen excretion per kg of BW gain decreased with reduced CP content (Figure 1b), from 2.1 to 2.9 g/kg BW gain/CP percentage point (Figure 1b). The analytical results of manure composition are presented in Figure 2a. Nitrogen and moisture content decreased slightly when the dietary CP content was reduced. The calculated nitrogen volatilization also decreased when dietary CP content was reduced (−3.9 to −6.4 points/CP percentage point; Figure 2b).

## Discussion

To investigate the effects of reducing the dietary CP content in broiler feed, two successive experiments were performed. Using an adjusted AA profile to test the response to the decrease in dietary CP, no significant effect was found between treatments for BW, BW gain and feed intake in experiment 1, while FCR was maintained until 17% CP and increased below this level. In experiment 2, reducing the dietary CP content did not affect FCR, even with 16% CP. This means that implementing the adjusted ideal AA profile of Mack *et al*. (1999) with changes in dArg:dLys and dThr:dLys ratios allows to decrease the CP content by 2 to 3 percentage points in broiler diets without impairing performance. These findings contradict the decrease in BW gain and feed efficiency frequently described in the literature (Alleman and Leclercq, 1997; Bregendahl *et al*., 2002; Dean *et al*., 2006), even though the indispensable AA requirements, in these studies, were apparently met. This negative effect on performance was also reported in a meta-analysis modelling the responses of BW gain and feed efficiency on dietary CP content (Pesti, 2009). In the present study, BW gain was unchanged and the negative impact of the reduction in CP content on FCR was found to be lower than in this meta-analysis (−0.017 *v*. −0.033 per 1% CP). The improvement in feed efficiency by genetic selection of broilers during this last decade could be an explanation to this difference, as Pesti’s study was based on trials performed before 2007. The reduction in dietary CP content tended to result in an increase in feed intake in experiment 2 (*P*=0.053). One hypothesis might be that the reduction of dietary CP is associated with a reduction of some indispensable AA such as Leu that, when provided in excess, are known to limit ingestion in various species including pigs (Gloaguen *et al*., 2012). Another explanation might be that broilers consumed more feed because they needed more protein to attain their genetic potential according to the theory of feed intake and growth proposed by Emmans (1987). Nevertheless, more recent findings reported by Gous (2007) did not fully confirm this theory since, instead of increasing feed intake as dietary CP content was reduced, some broiler genotypes reduced their feed intake. The possibility of broilers to adjust feed intake according to their genotype has implications for the optimization of feeding strategies. An increase in feed intake (without an increase in weight gain) would also partly explain the increase in abdominal fat such as the one we observed with low CP diets, the excess of energy being stored as fat.

This significant increase in abdominal fat with reduced CP content is in agreement with the literature, whereas the findings are more controversial for breast meat yield that remained unchanged (with a tendency for an increase) but decreased according to other studies (Alleman and Leclercq, 1997; Berres *et al*., 2010; Namroud *et al*., 2010). The increase in abdominal fat could here be explained by the increase of the dietary ME:CP ratio when reducing CP (Table 1). This suggests that the use of an adapted AA profile allows maximization of breast meat yield, at least until 16% CP. In addition, meat quality traits measured in experiment 2 were affected by the reduction in dietary CP content. It resulted in an increase in ultimate pH of breast meat. According to several authors, the ultimate pH is negatively correlated to the glycogen content of the muscle (Berri *et al*., 2008, Le Bihan-Duval *et al*., 2008). We assumed that the provision of a more balanced AA profile (associated with the reduction in CP content) reduced the excess of AA, decreasing the amount of nutrients available for glycogen production/storage through AA catabolism. Several studies have indeed shown that the ultimate pH responds to dietary treatments and in particular to variation in the Lys, Met or protein content (Berri *et al*., 2008; Jlali *et al*., 2012; Conde-Aguilera *et al*., 2016). Moreover, we showed that the increase in ultimate pH of breast meat was associated with a lower lightness and drip loss, in agreement with results from others (Berri *et al*., 2008; Le Bihan-Duval *et al*., 2008). Moreover, as the ultimate pH increased with decreasing CP content, one can expect an improvement in the processing ability of breast meat, in line poultry processors’ expectations (Alnahhas *et al*., 2014).

The estimated nitrogen efficiency in the two experiments was improved by 3.2 to 3.6 percentage points/percentage point CP reduction. As shown in Table 4, these values are higher than those reported in the literature (up to +2 percentage points/percentage point CP reduction; Aletor *et al.,* 2000; Bregendahl *et al*., 2002; Gomide *et al*., 2011). Moreover, the efficiency of nitrogen retention for broilers that received diets with <16% CP were above 70%. Even though these values are extremely high and could be questioned, efficiencies >70% have been reported in growing broilers (Siegert *et al*., 2016). Further investigations are required to evaluate the physiological limit to the increase in the efficiency of nitrogen utilization in relation to the maximum metabolic utilization of absorbed AA. Two hypotheses can be formulated to explain the higher nitrogen retention found in our study. First, due to an adequate AA profile in our experimental diets, BW gain was maintained whereas growth and FCR were negatively impacted in other studies. Second, the broilers used in our study may have been more efficient, due to the genetic selection for feed efficiency for over more than a decade.

**Table 4:** Linear regressions of nitrogen retention efficiency (N_effi_) and nitrogen excretion (N_exc_BWG_) *v*. CP content from data published in the literature

				N_effi_				N_exc_BWG_			
References	Experiment	CP range	Age of broilers (days)	Slope	Intercept	RMSE	*R*²	Slope	Intercept	RMSE	*R*²
Bregendahl *et al*. (2002)	1	23.5 to 19	7 to 21	−1.50	86.77	0.49	0.96	1.34	−6.89	0.29	0.98
Bregendahl *et al*. (2002)	2	24 to 18.5	7 to 21	−1.23	84.51	1.19	0.78	1.14	−4.61	0.27	0.98
Bregendahl *et al*. (2002)	3	23.5 to 18.5	7 to 21	−1.40	87.30	0.59	0.92	1.33	−8.53	0.34	0.97
Aletor *et al.* (2000)	1	22.5 to 16	21 to 42	−2.02	92.92	1.57	0.87	1.90	−9.70	1.74	0.83
Gomide *et al*. (2011)	1	21 to 18	14 to 21	−1.67	105.40	0.67	0.83	1.69	−16.89	0.56	0.88
Gomide *et al*. (2011)	2	20 to 17	28 to 35	−1.44	92.55	0.63	0.80	2.04	−16.34	0.77	0.85

The main consequence of the improvement of the efficiency of nitrogen utilization was the reduction in nitrogen excretion as shown in Figure 1b. As for N_effi_, the reduction in excretion (−2.1 to −2.9 g/kg BW gain) was higher in the present experiments than in previous studies (Table 4), which can be explained by a higher efficiency of nitrogen utilization found in our experiment. Between 19% and 16% CP, the reduction of one CP percentage point decreased nitrogen excretion by 13% (Supplementary Table S5 for details on this calculation), while in the literature the decrease was closer to 10% (Aletor *et al.*, 2000; Bregendahl *et al*., 2002; Gomide *et al*., 2011). Moreover, reducing the dietary CP content decreased the nitrogen and moisture content of the manure, which also confirms previous reports on manure and fresh droppings (Ferguson *et al*., 1998; Si *et al*., 2004; Hernández *et al*., 2012). This reduction in moisture content may be due to a lower water intake and water excretion by broilers fed the low CP diets (Alleman and Leclercq, 1997; Hernández *et al.*, 2012). In our experiments, the decrease in dietary CP was associated with a lower use of soyabean meal, which is very rich in potassium. A decreased potassium content in the diet can lead to a lower water intake (Alleman and Leclercq, 1997; Francesch and Brufau, 2004).

The observed decrease in manure moisture content could also explain the lower volatilization rate of excreted nitrogen in pens with broilers fed low CP diets (−5 points/CP percentage point on average for the two experiments). The moisture content of manure is one of the main factors contributing to the microbial activity in manure, and thus in the transformation of excreted nitrogen into ammonia (Méda *et al*., 2011). This is in agreement with the work of Ferguson *et al*. (1998), who reported a decrease of moisture content and nitrogen volatilization in manure when the dietary CP content was reduced. As a consequence, the simultaneous decrease of nitrogen excretion and nitrogen volatilization percentage leads to strong and synergistic decrease of nitrogen amounts lost through volatilization by about −30%/CP percentage point (Supplementary Table S6 for details on this calculation).

## Conclusions

In conclusion, the present findings clearly show that a reduction of the dietary CP content by several CP percentage points is possible in growing-finishing broilers with positive implications for the sustainability of broiler production. By using the ideal AA profile of Mack *et al*. (1999) modified for Arg and Thr, growth performance of modern growing-finishing broilers was not affected when the diets provided between 19% and 17% CP. Such a nutritional strategy also had a positive impact on meat quality traits with potential better processing abilities for breast meat, and on the environment with reduced nitrogen excretion and volatilization rates. In the future, other nutritional strategies have to be identified to achieve an even greater decrease in dietary CP (i.e. below 17%). Also, the requirements for the next limiting AA (e.g. Val and Ile, and also non-indispensable AA such as Gly) should be assessed.

## Supplementary material

For supplementary material accompanying this paper visit http://dx.doi.org/10.1017/S1751731117000660. click here to view supplementary material
