# Supplementary material for: Reducing the CP content in broiler feeds: impact on animal performance, meat quality and nitrogen utilization
Source: Animal. 2017 May 2;11(11):1881–9. doi: 10.1017/S1751731117000660 (PMC5645801; doi:10.1017/S1751731117000660)
Supplement: Supplementary file 1 [file S1751731117000660sup001.docx]

**Supplementary materials**

**Reducing the crude protein content in broiler feeds: Impact on animal performance, meat quality, and nitrogen utilization**

**P. Belloir^1,2^, B. Méda^1^, W. Lambert^2^, E. Corrent^2^, H. Juin^3^, M. Lessire^1^, S. Tesseraud^1^**

*^1^URA, INRA, 37380 Nouzilly, France*

*^2^AJINOMOTO EUROLYSINE S.A.S., F-75817 Paris Cedex 17, France*

*^3^EASM, INRA, 17700 Saint-Pierre-d'Amilly, France*

^§^ Supported in part by Ajinomoto Eurolysine S.A.S. Research Grants.

* Corresponding author: bertrand.meda@inra.fr

Tel +33 2 47 42 78 47, Fax. +33 2 47 42 77 78

**Short title: Crude protein reduction in broiler diets**

**Table S1.** Feedstuffs and chemical composition (%) of the pre-experimental diets fed to Ross PM3 broilers.

|  | Experiment 0 (Supp. Mat) | |  | Experiments 1 & 2 (Main paper) | |
| --- | --- | --- | --- | --- | --- |
|  | Starter  (day1 to day6) | Grower  (day7 to day13) |  | Starter  (day1 to day9) | Grower  (day10 to day20) |
| Feedstuffs |  |  |  |  |  |
| Maize | 42.8 | 46.0 |  | 42.8 | 46.0 |
| Soybean meal (48% CP) | 38.6 | 31.8 |  | 38.6 | 31.8 |
| Wheat | 10.0 | 12.0 |  | 10.0 | 12.0 |
| Soybean oil | 4.4 | 5.0 |  | 4.4 | 5.0 |
| Maize gluten meal | - | 1.7 |  | - | 1.7 |
| Dicalcium phosphate | 2.3 | 2.0 |  | 2.3 | 2.0 |
| Calcium carbonate | 0.4 | 0.2 |  | 0.4 | 0.2 |
| Mineral-Vitamin premix^1^ | 0.9 | 0.9 |  | 0.9 | 0.9 |
| DL-Methionine | 0.4 | 0.3 |  | 0.4 | 0.3 |
| L-Lysine-HCl | 0.2 | 0.2 |  | 0.2 | 0.2 |
| L-Threonine | 0.1 | 0.1 |  | 0.1 | 0.1 |
| Calculated composition^2,3^ |  |  |  |  |  |
| AMEn(MJ/kg) | 12.6 | 13.2 |  | 12.6 | 13.2 |
| Crude protein | 23.6 | 21.7 |  | 23.1 | 21.3 |
| Calcium | 1.05 | 0.90 |  | 1.05 | 0.90 |
| Available phosphorus^4^ | 0.50 | 0.45 |  | 0.50 | 0.45 |
|  |  |  |  |  |  |
| Total Lysine | 1.39 | 1.23 |  | 1.37 | 1.21 |
| Total Met | 0.72 | 0.61 |  | 0.70 | 0.59 |
| Total Met+Cys | 1.06 | 0.93 |  | 1.05 | 0.93 |
| Total Trp | 0.28 | 0.25 |  | 0.28 | 0.24 |
| Total Thr | 0.94 | 0.87 |  | 0.93 | 0.86 |
|  |  |  |  |  |  |
| dLys | 1.27 | 1.13 |  | 1.26 | 1.11 |
| dMet | 0.69 | 0.58 |  | 0.68 | 0.57 |
| dMet+Cys | 0.99 | 0.97 |  | 0.98 | 0.86 |
| dThr | 0.85 | 0.78 |  | 0.83 | 0.77 |
| dTrp | 0.25 | 0.22 |  | 0.25 | 0.22 |

^1^Supplied per kilogram of diet: NaCl = 3g; Co = 0.6 mg; Cu = 20 mg; Fe = 58 mg; I = 2 mg; Mn = 80 mg; Se = 0.2 mg; Zn = 90 mg; retinyl acetate = 15000 IU; cholecalciferol = 4300 IU; DL-alpha tocopheryl acetate = 100 mg; thiamine mononitrate = 5 mg; riboflavin = 8 mg; calcium pantothenate = 25 mg; cyanocobalamin = 0.02 mg; menadione = 5 mg; pyridoxine hydrochloride = 7mg; folic acid = 3 mg; biotin = 0.3 mg; niacin = 100 mg; choline chloride = 550 mg; antioxidant (buthylhydroxyanisole, propyl gallat, ethoxyquin) = 50 mg.

^2^ Protein content was estimated from analysed CP content of feedstuffs.

^3^ Digestible amino acid content was calculated from total AA feedstuff content (chemical analyses) using digestibility coefficients from Sauvant *et al.* (2004).

^4^ Available phosphorus was calculated from total P feedstuff contents and availability coefficients from Sauvant *et al.* (2004).

**Table S2.** Experimental protocol and main results for experiment 0, carried out prior to experiments 1 and 2 (main paper). The objective was to investigate the possibility of decreasing dietary CP content in broilers from 14 to 21 days.

*Experimental design*

One hundred fifty day-old Ross PM3 male chicks from a commercial hatchery were reared together in an experimental poultry unit (INRA PEAT, Nouzilly, France) from day 1 to day 13. Birds were fed a starter and a grower diet (Table S1). They were individually wing-tagged at day 7. At day 14, 64 birds (405 ± 24g) were randomly assigned to two experimental diets (32 birds per treatment) and housed in individual cages. During the whole experiment, birds were watered and fed *ad libitum*. From day 14 to day 31 (experimental period), two experimental diets (Table S3) were formulated to be iso-energetic at 13.2 MJ ME/kg, to be sub-limiting in digestible lysine (dLys) at 0.9% (to avoid any other factor being limiting than CP) and to contain either 19% or 17% CP content by respecting the minimum AA:Lys ratios of the ideal AA profile proposed by Mack *et al.* (1999). The feedstuffs were analyzed before formulation, and diets were pelleted. For experimental diets, total theoretical and analysed AA contents were similar (Table S4).

*Performance and carcass characteristics (N=32 per treatment)*

|  | Diets | |  |  |
| --- | --- | --- | --- | --- |
|  | CP19% | CP17% | SEM | *P-*value |
| Performance | | | | |
| BW at day 31 (g) | 1818 | 1800 | 14.2 | 0.52 |
| BW gain (g) | 1411 | 1394 | 12.4 | 0.41 |
| Feed intake (g) | 2265 | 2292 | 20.6 | 0.52 |
| Feed conversion ratio | 1.60 | 1.64 | 0.01 | 0.056 |
| Carcass characteristics | | | | |
| Breast meat yield (% of BW) | 18.4^b^ | 19.1^a^ | 0.1 | 0.009 |
| Abdominal fat (% of BW) | 2.19^b^ | 2.36^a^ | 0.04 | 0.043 |

**Table S3.** Feedstuffs and chemical composition (%) of the diets differing in crude protein contents fed to Ross PM3 broilers housed in individual cages between 14 and 31 days of age (Experiment 0).

| Diets | CP19% | CP17% |
| --- | --- | --- |
| Feedstuffs |  |  |
| Maize | 46.9 | 53.9 |
| Soybean meal | 28.5 | 22.2 |
| Wheat | 15.0 | 15.0 |
| Soybean oil | 5.2 | 4.1 |
| Dicalcium phosphate | 1.7 | 1.7 |
| Calcium carbonate | 1.1 | 1.1 |
| Elancoban 200 | 0.05 | 0.05 |
| Mineral-Vitamin premix^1^ | 0.80 | 0.80 |
| DL-Methionine | 0.16 | 0.22 |
| L-Lysine-HCl | 0.04 | 0.22 |
| L-Threonine | - | 0.04 |
| L-Arginine | - | 0.07 |
| L-Valine | - | 0.03 |
| Calculated composition |  |  |
| AMEn (MJ/kg) | 13.2 | 13.2 |
| Crude protein | 19.0 | 17.0 |
| Calcium | 1.00 | 1.00 |
| Available phosphorus^2^ | 0.40 | 0.40 |
| Calculated digestible AA values^3,4^ |  |  |
| dLys | 0.90 | 0.90 |
| dMet | 0.41 (46) | 0.44 (49) |
| dMet+Cys | 0.67 (74) | 0.67 (74) |
| dThr | 0.62 (69) | 0.57 (63) |
| dVal | 0.81 (90) | 0.73 (81) |
| dIle | 0.74 (82) | 0.63 (70) |
| dLeu | 1.48 (164) | 1.34 (149) |
| dTrp | 0.20 (22) | 0.17 (19) |
| dArg | 1.1 (122) | 1.01 (112) |
| dPhe | 0.87 (97) | 0.73 (81) |
| dTyr | 0.62 (69) | 0.54 (60) |
| dPhe+Tyr | 1.49 (166) | 1.27 (141) |
| dHis | 0.44 (49) | 0.39 (43) |
| dSer | 0.84 (91) | 0.73 (79) |
| dGly | 0.66 (71) | 0.57 (62) |
| dSer+Gly | 1.50 (163) | 1.31 (141) |

^1^ Supplied per kilogram of diet: NaCl = 3g; Co = 0.6 mg; Cu = 20 mg; Fe = 58 mg; I = 2 mg; Mn = 80 mg; Se = 0.2 mg; Zn = 90 mg; retinyl acetate = 15000 IU; cholecalciferol = 4300 IU; DL-alpha tocopheryl acetate = 100 mg; thiamine mononitrate = 5 mg; riboflavin = 8 mg; calcium pantothenate =25 mg; cyanocobalamin = 0.02 mg; menadione = 5 mg; pyridoxine hydrochloride = 7mg; folic acid = 3 mg; biotin = 0.3 mg; niacin = 100 mg; choline chloride = 550 mg; antioxidant (buthylhydroxyanisole, propyl gallat, ethoxyquin) = 50 mg.

^2^ Available phosphorus was calculated from total P feedstuff contents and availability coefficients from Sauvant *et al.* (2004).

^3^ Digestible amino acid content was calculated from total AA feedstuff content (chemical analyses) using digestibility coefficients from Sauvant *et al.* (2004).

^4^ dAA:Lys ratios (%) are given between brackets. In all diets, dAA:dLys ratios were equal or above the ratios proposed by Mack *et al.* (1999): dMet+Cys:Lys=75, dThr:dLys=63, dVal:dLys=81, dIle:dLys =71, dTrp:dLys=108, dArg:dLys=112.

**Table S4.** Crude protein and total amino acid content (%) for all diets differing in crude protein contents.

| Experiment | Experiment 0 | |  | Experiment 1 | | | | |  | Experiment 2 | | |
| --- | --- | --- | --- | --- | --- | --- | --- | --- | --- | --- | --- | --- |
| Diets | 19% | 17% |  | 19% | 18% | 17% | 16% | 15% |  | 19% | 17.5% | 16% |
| Analysed Crude protein | 19.4 | 17.7 |  | 19.1 | 18.1 | 17.1 | 15.6 | 15.0 |  | 19.1 | 17.4 | 16.1 |
| Theoretical total AA values |  |  |  |  |  |  |  |  |  |  |  |  |
| Total Lys | 1.00 | 0.98 |  | 1.00 | 0.99 | 0.98 | 0.97 | 0.97 |  | 1.00 | 0.98 | 0.97 |
| Total Met | 0.44 | 0.46 |  | 0.43 | 0.44 | 0.45 | 0.46 | 0.48 |  | 0.43 | 0.44 | 0.46 |
| Total Met+Cys | 0.73 | 0.73 |  | 0.74 | 0.73 | 0.73 | 0.72 | 0.72 |  | 0.74 | 0.73 | 0.72 |
| Total Trp | 0.23 | 0.19 |  | 0.23 | 0.22 | 0.21 | 0.20 | 0.19 |  | 0.23 | 0.2 | 0.19 |
| Total Thr | 0.69 | 0.63 |  | 0.69 | 0.68 | 0.68 | 0.67 | 0.67 |  | 0.69 | 0.68 | 0.67 |
| Total Leu | 1.59 | 1.43 |  | 1.53 | 1.44 | 1.35 | 1.26 | 1.18 |  | 1.53 | 1.42 | 1.27 |
| Total Ile | 0.80 | 0.69 |  | 0.77 | 0.75 | 0.73 | 0.70 | 0.68 |  | 0.77 | 0.70 | 0.69 |
| Total Val | 0.89 | 0.80 |  | 0.87 | 0.85 | 0.83 | 0.81 | 0.79 |  | 0.87 | 0.80 | 0.79 |
| Total Arg | 1.20 | 1.09 |  | 1.19 | 1.15 | 1.11 | 1.07 | 1.03 |  | 1.19 | 1.06 | 1.04 |
| Total Phe | 0.94 | 0.81 |  | 0.92 | 0.85 | 0.78 | 0.71 | 0.64 |  | 0.92 | 0.83 | 0.71 |
| Total Tyr | 0.67 | 0.58 |  | 0.66 | 0.61 | 0.56 | 0.51 | 0.46 |  | 0.66 | 0.60 | 0.51 |
| Total His | 0.48 | 0.42 |  | 0.49 | 0.45 | 0.42 | 0.38 | 0.35 |  | 0.49 | 0.44 | 0.39 |
| Total Ser | - | - |  | 0.90 | 0.83 | 0.76 | 0.69 | 0.62 |  | 0.90 | 0.81 | 0.70 |
| Total Gly | - | - |  | 0.77 | 0.71 | 0.65 | 0.59 | 0.54 |  | 0.77 | 0.70 | 0.60 |
| Analyzed total AA values |  |  |  |  |  |  |  |  |  |  |  |  |
| Total Lys | 1.06 | 1.03 |  | 1.02 | 1.03 | 1.04 | 1.01 | 1.03 |  | 1.02 | 1.02 | 1.02 |
| Total Met | 0.44 | 0.45 |  | 0.42 | 0.41 | 0.44 | 0.41 | 0.47 |  | 0.42 | 0.43 | 0.45 |
| Total Met+Cys | 0.74 | 0.72 |  | 0.30 | 0.28 | 0.27 | 0.25 | 0.24 |  | 0.73 | 0.71 | 0.70 |
| Total Trp | 0.24 | 0.20 |  | 0.23 | 0.22 | 0.21 | 0.19 | 0.19 |  | 0.23 | 0.20 | 0.19 |
| Total Thr | 0.72 | 0.66 |  | 0.69 | 0.69 | 0.69 | 0.66 | 0.67 |  | 0.69 | 0.69 | 0.68 |
| Total Leu | 1.61 | 1.45 |  | 1.52 | 1.46 | 1.39 | 1.25 | 1.18 |  | 1.52 | 1.42 | 1.28 |
| Total Ile | 0.82 | 0.70 |  | 0.77 | 0.77 | 0.76 | 0.71 | 0.69 |  | 0.77 | 0.70 | 0.70 |
| Total Val | 0.92 | 0.82 |  | 0.86 | 0.87 | 0.86 | 0.81 | 0.80 |  | 0.86 | 0.80 | 0.80 |
| Total Arg | 1.24 | 1.12 |  | 1.17 | 1.17 | 1.16 | 1.07 | 1.06 |  | 1.17 | 1.06 | 1.04 |
| Total Phe | 0.97 | 0.84 |  | 0.91 | 0.86 | 0.81 | 0.7 | 0.65 |  | 0.91 | 0.83 | 0.72 |
| Total Tyr | 0.69 | 0.59 |  | 0.65 | 0.60 | 0.56 | 0.49 | 0.45 |  | 0.65 | 0.60 | 0.52 |
| Total His | 0.49 | 0.43 |  | 0.47 | 0.46 | 0.43 | 0.38 | 0.35 |  | 0.47 | 0.44 | 0.39 |
| Total Ser | - | - |  | 0.91 | 0.85 | 0.79 | 0.69 | 0.65 |  | 0.91 | 0.83 | 0.73 |
| Total Gly | - | - |  | 0.77 | 0.72 | 0.68 | 0.59 | 0.55 |  | 0.77 | 0.70 | 0.61 |

**Table S5.** Effect of one percentage point CP decrease on nitrogen excretion.

Using the specific linear regressions in Figure 1b, amounts of N excreted (N_exc_, g/kg BW gain) for CP contents between 19 and 16% were calculated.

| **Experiment 1** | | |
| --- | --- | --- |
| CP (%) | N_exc_ | Variation in N_exc_ |
| 19 | 19.4 |  |
| 18 | 17.2 | (17.2 - 19.4) / 19.4 = -11% |
| 17 | 15.1 | (15.1 - 17.2) / 17.2 = -12% |
| 16 | 13.0 | (13.0 - 15.1) / 15.1 = -12% |
| **Average = -12%** | | |
| **Experiment 2** | | |
| CP (%) | N_exc_ | Variation in N_exc_ |
| 19 | 24.1 |  |
| 18 | 21.3 | (21.3 - 24.1) / 24.1 = -12% |
| 17 | 18.4 | (18.4 - 21.3) / 21.3 = -13% |
| 16 | 15.6 | (15.6 - 18.4) / 18.4 = -15% |
| **Average = -14%** | | |
| **Overall average = -13%** | | |

**Table S6.** Effect of one percentage point CP decrease on amounts of volatilized nitrogen.

Using the specific linear regressions in Figure 1b and Figure 2b, amounts of N excreted (N_exc_, g/kg BW gain) and % of volatilized nitrogen (N_vol_, % of N_exc_) were calculated for CP contents between 19 and 16%. Amounts of volatilized nitrogen were then calculated (g N lost / kg BW gain)

| **Experiment 1** | | | | |
| --- | --- | --- | --- | --- |
| CP (%) | N_exc_ | N_vol_ | Volatilized N (g/kg BW gain) | Variation |
| 19 | 19.4 | 27.5 | 5.3 |  |
| 18 | 17.2 | 23.6 | 4.1 | -24% |
| 17 | 15.1 | 19.7 | 3.0 | -27% |
| 16 | 13.0 | 15.8 | 2.0 | -31% |
| **Average = -27%** | | | | |
| **Experiment 2** | | | | |
| CP (%) | N_exc_ | N_vol_ | Volatilized N (g/kg BW gain) |  |
| 19 | 24.1 | 36.1 | 8.7 |  |
| 18 | 21.3 | 29.6 | 6.3 | -28% |
| 17 | 18.4 | 23.2 | 4.3 | -32% |
| 16 | 15.6 | 16.7 | 2.6 | -39% |
| **Average = -33%** | | | | |
| **Overall average = -30%** | | | | |
